# False Reports from Patients with Frontotemporal Dementia: Delusions or Confabulations?

**DOI:** 10.3233/BEN-2011-0335

**Published:** 2011-08-29

**Authors:** Mario F. Mendez, Ivan Andrew Fras, Sarah A. Kremen, Po-Heng Tsai

**Affiliations:** Departments of Neurology and Psychiatry and Biobehavioral SciencesDavid Geffen School of MedicineThe University of California at Los AngelesV.A. Greater Los Angeles Healthcare CenterLos AngelesCAUSA

## Abstract

Patients with behavioral variant frontotemporal dementia (bvFTD) can make false statements consistent with delusions or confabulations. It is unclear whether bvFTD is primarily associated with either delusions or with confabulations and whether they can be explained by the pathophysiology of this disease. In order to clarify this, we retrospectively surveyed the records of 48 patients with bvFTD for the presence of any false reports and identified four patients. Their false reports included continued interaction with a favorite but dead relation, fictitious marriages with movie stars, and two who claimed that their partner was having an affair. When confronted with the falsity of their statements, the patients conveyed a lack of certainty regarding their external or internal source but persisted in the constancy of their reports. On functional neuroimaging, the patients had predominant frontal involvement. This report found that patients with bvFTD can have both fantastic, wish fulfilling confabulations and typical content-specific delusions. We propose that both phenomena result from known disturbances of ventromedial prefrontal cortex in bvFTD, including deficits in source monitoring and in activating an automatic “doubt tag” for false reports.

## 1. Introduction

Behavioral variant frontotemporal dementia (bvFTD) is a neurodegenerative disorder with a usual onset in the presenium. It is pathologically heterogeneous with abnormal intraneuronal deposits of misfolded proteins including tau, TDP-43, or fused-in-sarcoma (FUS) [23, 47]. Clinically, bvFTD presents as alterations in social behavior and in related behavioral and personality changes. The criteria for bvFTD include apathy or inertia, social disinhibition, eating disorders, compulsive or repetitive behaviors, loss of empathy or sympathy, and frontal executive deficits with relative preservation of memory and visuospatial skills [23,42]. Some patients with bvFTD also make false statements highly suggestive of delusions or confabulations.

The presence of delusions or confabulations in bvFTD is intriguing because of the potential underlying mechanisms and, in the case of delusions, the clinical similarities to schizophrenia [32,45,56]. False reports could be confabulations, delusions, delusional memories, or "fantastic thinking" [4,26]. It is unclear, however, whether false statements by bvFTD patients are most commonly delusions or confabulations and whether they can be explained by known mechanisms of dysfunction in bvFTD. Although the incidence of delusions and hallucinations in bvFTD is low [10,36], delusions particularly occur among those with very early onset bvFTD and those with accompanying motor neuron disease (FTD-MND) [31,58]. Other investigators indicate that false reports among bvFTD patients are actually confabulations or a variant of spontaneous confabulations described as "fantastic thinking" [26, 43].

Delusions and confabulations have similarities and differences. A delusion is a fixed belief that is false but firmly held despite all evidence to the contrary [6, 8,25]. In contrast, typical provoked confabulations are memory-related misstatements retrieved out of tem-

ISSN 0953-4180/11/$27.50 © 2011 – IOS Press and the authors. All rights reserved poral context [25]. Confabulations usually occur in amnestic disorders such as Wernicke-Korsakoff's syndrome and focal frontal or limbic lesions or encephalitides [3,4]. Both delusions and confabulations are associated with frontal-executive deficits and deficiencies in self-monitoring [21,24]. The main differences between delusions and confabulations are that the former involve belief-formation, are systematic or pervasive, and more fixed, whereas the latter involve memory-retrieval deficits, are isolated or fleeting, and more variable [10,17,27]. Some patients, however, may have spontaneous confabulations that are implausible and indistinguishable from delusions [15], suggesting an overlap between the two and a possible common pathophysiology.

We surveyed a population of patients with bvFTD in order to determine whether they had primarily delusions, confabulations, or some overlap between the two. We then analyzed the clinical aspects of their false beliefs in order to see if they could be explained by known mechanisms of frontal dysfunction, particularly reported deficits in ventromedial prefrontal cortex (VMPFC). This study analyzes the nature of their false reports for the characteristic features of delusions or confabulations. Confabulatory reports and delusions both occurred in these patients. We conclude with a discussion of the possible implications for understanding these phenomena in among patients with bvFTD.

## 2. Case reports

The records of 48 patients seen in the UCLA Neurobehavior Clinic between 2006–2010 and meeting diagnostic criteria for bvFTD were retrospectively reviewed for the presence of false reports. Inclusion criteria included bvFTD established by diagnostic criteria and the presence, by caregiver report, of persistent or recurrent statements known to be false. Exclusion criteria included evidence of an identifiable alternative explanation for the false statements, such as misunderstandings, misperceptions, or another potentially causative neuropsychiatric condition. This review identified four bvFTD patients with recorded false reports. All four had clinical and neurobehavioral evaluations, brain magnetic resonance imaging (MRI), and functional neuroimaging (See Table 1). The prevalence of false reports in this population was only 8.3%, but this patient population does not include FTD-MND, who may have more delusions [31]. Patient No. 2 was previously reported with pathologically confirmed Pick's disease [26].

### *2.1. Patient No. 1.*

A 48-year-old left-handed man had a 3-year history of an insidious and progressive personality change which began with difficulty completing tasks and initiating interpersonal interactions. He also became disinhibited, making inappropriate comments and touching others. He was emotionally detached from his family, disheveled in his personal appearance, and compulsively hoarded stacks of magazines and other items. The patient developed particular food desires, including ice cream cones at 8 o'clock in the morning, ice cream for lunch, and peanut butter and jelly sandwiches at night. Past medical history was positive for thyroid disease and bipolar disease with a single episode of depression and a fluoxetine-induced manic episode. His family history was negative for bipolar disease, dementia, or any neurological diseases.

Of great concern to his family was the additional development of false reports. He continued to tell them that he was interacting with his ex-father-in-law who had died six years previously. The patient, who had had a close relationship with his former father-in-law, reported ongoing conversations and meetings with him. When confronted with his death, the patient acknowledged that his ex-father-in-law had died many years previously but concluded that he must have returned from death somehow. He would then persist in his report of these ongoing interactions but admitted that it was possible that it was not really happening outside of his head (See Table 2). On subsequent interviews, he would again report these ongoing interactions.

On examination, he had a flat affect with a tendency to be detached from the conversation. He stated that nothing was wrong with him. His neurobehavioral examination showed deficits on tests of executive functions (See Table 1) [7,9]. Language was fluent, with good auditory comprehension, repetition, and confrontational naming. Reading and writing were adequate, except for his tendency to be stuck on the literal aspects of the task, often repeating the whole task instructions. His knowledge of current events and historical events was excellent. The patient performed normally on ideomotor praxis tasks. His neurological examination, including cranial nerves, coordination, motor, reflex, and sensory testing, was normal except for slight bilaterally increased extrapyramidal tone.

The diagnostic work-up was consistent with bvFTD. His MRI scan showed clear and disproportionate frontal atrophy with compensatory enlargement of the frontal horns, and positron emission tomography (PET)

Table 1
Characteristics of patients

| | Patient 1 | Patient 2 | Patient 3 | Patient 4 |
|---|---|---|---|---|
| Age at onset | 45 | 50 | 44 | 51 |
| Age at presentation | 48 | 53 | 50 | 53 |
| Sex | M | F | M | M |
| Education (years) | 16 | 12 | 12 | 14 |
| Mini-Mental State Examination (30) | 21 | 30 | 30 | 26 |
| Digit Span (Normal $\geqslant$ 5)[A] | 6 | 6 | 6 | 5 |
| Verbal Fluency: | | | | |
| Animals/minute (Normal $\geqslant$ 12)[A] | 18 | 10 | 5 | 11 |
| "F" words/minute (Normal $\geqslant$ 10)[A] | 13 | 7 | 8 | 6 |
| Mini-Boston Naming Test (15) | 15 | 15 | 15 | 11 |
| CERAD | | | | |
| Delayed Recall) (Normal $\geqslant$ 7/10)[A] | 3 | 4 | 2 | 1 |
| Recognition (20) (Normal $\geqslant$ 16/20)[A] | 9 (0FP) | 9 (0FP) | 5 (5FP) | 10 (3FP) |
| Visuospatial (8) | 8 | 8 | 7 | 7 |
| Frontal Assessment Battery (18)[B] | 12 | 11 | 11 | 10 |
| Delis-Kaplan Proverbs (8)[B] | 2 | 6 | 0 | 1 |
| Functional Neuroimaging | Bifrontal, including VMPFC | right > left frontal including VMPFC | bifrontal- including VMPFC; bitemporal | left > right frontal including VMPFC |

CERAD = Consortium to Establish a Registry in Alzheimer's Disease; FP = false positives.
[A]Approximate normal cut-off scores for these four patients;
[B]Frontal-executive tasks indicate $\leqslant$ 1 standard deviation below normal.

Table 2
Characteristics of false reports

| | Patient 1 | Patient 2 | Patient 3 | Patient 4 |
|---|---|---|---|---|
| Content | Persistent Interaction with Dead Relative | Many Celebrity Lovers | Spousal Infidelity | Spousal Infidelity |
| Implausible or Incomprehensible | Yes | Yes | Yes – wife next to him during affair | Yes – needed instantaneous travel to far site |
| Incorrigibility or Fixed Constancy | Yes | Yes | Yes | Yes |
| Certainty or Lack of Doubt | No – could be in his mind | No – could be imagination | No – could be in his mind | No – could be in his mind |
| Completion of Impaired Memories | No | No | No | No |
| Self-relevant or Self-important Content | Yes | Yes | Yes | Yes |
| Imagination or Wish fulfillment | Yes | Yes | No | No |

showed decreased metabolic activity in the frontal lobes. The patient was managed with memantine only; his false reports were not targeted for therapy.

### 2.2. *Patient No. 2.*

A 53-year old woman had a 5–6 year history or personality and behavioral changes beginning with disengagement and withdrawal from social interactions and activities, emotional detachment, impulsivity, and decreased self-care. She began making inappropriate comments to strangers, including confronting them about their sexual orientation and smoking behavior. Her appetite changed with a tendency towards sweets, and she developed compulsive, repetitive, and stereotypical behaviors. Her past medical history and family history were otherwise negative for neurological disorders.

As part of her personality change, the patient reported relationships with famous actors. She reported interacting with them, marrying them, having sex with them, and, eventually, having children with them. When confronted with the implausibility of these relationships and the existence of her real husband, she replied that her real husband had gotten lost while hiking despite his presence next to her. When pressed on the nature of these relationships, she persisted in reported them, but did admit that they might not be real, that they were in her imagination, and that she was still married to her husband.

On examination, the patient had a fixed smile and frequent staring. She was alert, oriented, and attentive.

Her language was fluent with good repetition and auditory comprehension. She had intact knowledge of current and historical events. General neuropsychiatric observations were consistent with silly, puerile behavior. Her neurological examination, including cranial nerves, gait and station, motor and sensory testing, and reflexes, was otherwise within normal limits.

Her diagnosis was bvFTD. MRI revealed cerebral atrophy, out of proportion in the frontal lobes, and a single photon emission tomography showed hypoperfusion predominantly in the right temporal and frontal lobes. She received quetiapine for treatment of her false belief phenomena without benefit. Soon after, the patient began to deteriorate leading to death six months later. Autopsy revealed Pick's disease with severe neuronal loss, gliosis, and spongiosis of the deep cortical layers in both frontal and anterior temporal lobes, but greater on the left [26].

### *2.3. Patient No. 3.*

A 50-year-old right-handed man had a six year history of an insidious and progressive personality change with anxiety, irritability, and increased use or crude and profane language. He had progressive difficulty performing his usual activities and had decreased attention to his personal hygiene. He had increased oral behaviors including shoving whole packs of gum into his mouth and constantly smoking or eating sweets. The patient developed compulsive-like behaviors such as repeatedly cleaning his car. He began seeing snakes in his house behind the chest of drawers and in other places, and, at one point, he took out a gun and shot at a perceived snake. His past medical history was otherwise negative except for significant substance abuse involving cocaine, marijuana, and alcohol. His family history was negative for any familial illnesses.

Another behavioral symptom was the new belief that his wife was having an affair with his neighbor. He would constantly look out at his neighbor's window, even when his wife was at home, and he would describe the experience of his wife and neighbor having sex. He "could see them having sex in his mind." When confronted with the fact that his wife could not be having an affair since she was standing next to him, he replied that it was her ghost that was having the affair. Although he persisted in this belief, he conveyed doubt as to whether it was taking place in the external world or just in his head. Nevertheless, he subsequently threatened the neighbor with harm because of the continued belief in the neighbor's affair with his wife.

On examination, he stated, "my brain is losing cells fast and it is in my frontal lobes." He manifested anger with frequent outbursts primarily aimed at his neighbor. The language exam appeared fluent, and auditory comprehension was intact. His knowledge of current and historical events was adequate. Neuropsychiatric observations were consistent with the overt presence of anger. His cranial nerves, gait and motor examination, reflexes, and sensory testing were all within normal limits.

His diagnosis was bvFTD. His MRI showed mild volume loss but no focal lesions or changes, and his PET showed hypometabolism in the frontal and anterior temporal lobes bilaterally out of proportion to any other areas. He received quetiapine at 800 mg a day for treatment of his delusional jealousy without benefit.

### *2.4. Patient No. 4.*

A 53-year-old, right-handed man had a 1–2 year history of word-finding difficulties, memory problems, temper outbursts, and inability to perform complex tasks. He would sit at home most of the day without productive activities but was also disinhibited, readily talking to strangers or waving at them. He had become obsessed with putting the trash out as soon as there was anything in the container. He was also obsessed with calling 800 numbers and made 10–20 calls per day to his wife and to an out-of-state friend. The patient had recently begun chewing on his fingers and usually had tooth picks, mints, and other things in his mouth. Finally, the patient's appetite and eating changed with food preferences for hamburgers, French fries, and strawberry milkshakes and other sweets. More recently, he complained of an episode of seeing alligators at night, without any preceding or subsequent experiences of hallucinations. His past medical history was positive for gastroesophageal reflux and prior heavy alcohol use. The family history was negative for any relatives with dementia or neurological diseases.

As part of his personality change, the patient had become morbidly jealous of his wife, accusing her of having an affair with their real estate agent in another state. He was convinced that his wife was focused on this other man, even when she clearly could not have travelled to see him. When confronted with this, he rationalized that she could instantaneously travel across far distances to see him, but could not explain how. He persisted in the belief of his spouse's affair but explained that "thinking it is as good as knowing it."

On examination, the patient sat with a broad grin and showed very limited insight into the nature of his condition. On testing, language showed word-finding difficulties but intact comprehension and repetition. Throughout the interview the patient would become quite stuck on the terms that he used during prior moments in the interview. He had adequate knowledge of current and historical events. The patient's praxis testing was within normal limits, but he perseverated on these tasks. His neurological examination was intact for cranial nerves, gait, motor and sensory testing, and reflexes, except for a tendency to bite his fingernails and put his fingers in his mouth.

His presentation was consistent with bvFTD. An MRI was normal, but a PET scan showed marked bifrontal cortical hypometabolism, left greater than right, and probable mild bitemporal hypometabolism. The patient, who was previously on galantamine, was started on low dose risperidone and sertraline with little impact on his morbid jealousy.

## 3. Discussion

These patients presented with false reports associated with bvFTD. For two of these patients, the false reports were consistent with spontaneous confabulations with content reflecting wish fulfillment. For two others, there were content-specific delusions with morbid jealousy. All four patients expressed equivocation as to the external source of their reports but, nevertheless, persisted in these implausible statements. We conclude that both spontaneous confabulations and delusions occur in bvFTD and suggest that they result from the same frontal lobe disturbances in this disorder, specifically impairment of source monitoring coupled with attenuation of the preconscious feelings of doubt or "doubt tag" that something is not true [51].

Delusions can occur in bvFTD. Delusions are false beliefs that are incomprehensible, incorrigible, and held with subjective conviction despite proof or evidence to the contrary [27]. Although they are infrequent [10,36], they may occur in up to 14% of patients with bvFTD, particularly if they belong to specific subgroups [31,44,58]. Two of our patients have morbid jealousy or the Othello syndrome, a delusional belief that their spouse or sexual partner was unfaithful. Other bvFTD patients have had the delusion of pregnancy and de Clerambault syndrome, a delusional belief of being loved by someone else [29,49]. Other content-specific delusions, such as the Capgras syndrome, or the delusion of a familiar person being replaced by an imposter, and related misidentification syndromes, are rare in bvFTD [19,23], possibly because these patients had difficulty detecting personally salient information in others. In bvFTD, delusions tend to be associated with an early onset [58], as in our two patients, associated motor neuron disease (FTD-MND) [31], TDP-43 or FUS pathologies [33,55,57], and, possibly, presenilin 1 mutations [48]. In these conditions, the delusions may precede the dementia by many years [27,59] and may be associated with prodromal bipolar, schizotypical, or "schizophrenia" [30,58,59]. In one series, 5 of 17 patients with early onset bvFTD in their 40's or younger had presented with a psychotic illness an average of 5 years prior to the dementia diagnosis [58]. In bvFTD, reported psychotic phenomenology can be typically paranoid or religious but also includes somatic and infestation delusions and visual and tactile hallucinations [44].

In addition to delusions, spontaneous confabulations can occur in bvFTD [43]. Confabulation are "honest lying" for the purpose of creating a "coherent self-narrative" of oneself in time and in relation to the world [12]. The most common confabulations are provoked, momentary, simple, and plausible errors in content or temporal order which fill in memory gaps [3, 25,41]. In our bvFTD patients, the confabulations are spontaneous, fantastic, grandiose, or impossible statements elaborated upon without the need to fill in a memory gap [41]. Unlike provoked confabulations, spontaneous confabulations can be internally-generated ideas associated with vivid imagination, wish fulfillment, embellishment, and story-telling [13,22,26]. Two of our patients had spontaneous confabulations with a wish fulfillment theme. Patients with bvFTD confabulate significantly more than patients with Alzheimer's disease (AD) [43], and the presence of confabulations may help discriminate patients with bvFTD from those with AD [50].

There are similarities between delusions and confabulations [25,28]. Both have frontal-executive dysfunction with impairment in accurately evaluating the truth of retrieved or generated information [15,22,24]. Our patients commented that their false reports could be in their head, imagined, or consistent with "thinking is as good as knowing it." Although perceptual biases, cognitive predispositions, and other factors can contribute to equivocation about the external source of false reports, source monitoring deficits specifically impair the ability to distinguish real memories or events from internally-generated thoughts with a tendency to identify imagined events as externally driven [21,22, 25]. For example, in source monitoring experiments, confabulating patients exhibit a bias towards identifying imagined events, which may be wish fulfilling, as externally driven [22,52,53]. Several studies suggested that the main area implicated in source monitoring is the VMPFC [16], and source monitoring deficits are observed mainly in disorders affecting frontotemporal areas [11].

Another similarity between delusions and confabulations is an abnormal level of certainty for the false report [39]. Our patients continued to report their delusions or confabulations despite proof to the contrary. This suggests attenuation of the normal "doubt tag" or intuitive, immediate "feeling of rightness," an automatic, unconscious checking system for memories or thoughts [51]. Without these normal feelings of doubt, individuals may not go on to perform a conscious, slow, and effortful checking for the veracity of memories or thoughts [15,20,25,37,51]. In addition to disturbed source monitoring [15,16], the VMPFC also appears responsible for the normal "doubt tag" or decrease in feeling of rightness [1,16,40,51].

Much research supports participation of the VMPFC and related areas in delusions and confabulations [15, 16,52,53]. Whereas disturbances of limbic structures can lead to paranoia or delusions, patients with bvFTD with delusions have bilateral or predominant right-sided frontal atrophy [38,44], and other neurological patients with delusions have right hemisphere or bifrontal lesions [8]. Whereas amnestic states from bilateral hippocampal, mamillary body, or related injury can lead to momentary or provoked confabulations, most studies indicate that lesions in the VMPFC are sufficient for confabulation [2,5,16,53]. The posterior medial OFC may contribute to confabulations via an inability to suppress interference of thoughts or extinguish previous anticipations [14,15,25,41,46]. Functional MRI studies also point to prefrontal cortex, particularly right VMPFC, as involved in confabulations and false recollections [34], and false recognition correlates negatively with grey matter density in prefrontal areas [35]. VMPFC activity is associated with identifying whether previously imagined words were previously seen or imagined [54].

In conclusion, patients with bvFTD can have both fantastic confabulations and delusions. For the former, the content reflects wish fulfillment with elaborative characteristics and fanciful personal narratives [18]. Two others had the delusion of spousal infidelity. We propose that these phenomena may originate from similar VMPFC deficits in source monitoring coupled with attenuation of a "doubt tag." When challenged, conscious checking leads to a lack of certainty as to their external source. This hypothesized mechanism, suggested by an analysis of these four patients, is only preliminary. In order to establish this mechanism, investigators need to pursue detailed prospective research on delusions and confabulations among bvFTD patients. Nevertheless, this clinical case analysis can help point the way for future research and investigations in this area.

## References

[1] A. Abraham, H. Rakoczy, M. Werning, D.Y. von Cramon and R.I. Schubotz, Matching mind to world and vice versa: Functional dissociations between belief and desire mental state processing, *Soc Neurosci* **5**(1) (2010), 1–18.

[2] D.F. Benson, A. Djenderedjian, B.L. Miller, N.A. Pachana, L. Chang, L. Itti and I. Mena, Neural basis of confabulation, *Neurology* **46**(5) (1996), 1239–1243.

[3] N. Berlyne, Confabulation, *Br J Psychiatry* **120**(554) (1972), 31–39.

[4] S. Borsutzky, E. Fujiwara, M. Brand and H.J. Markowitsch, Confabulations in alcoholic Korsakoff patients, *Neuropsychologia* **46**(13) (2008), 3133–3143.

[5] C. M. Braun, J. Delisle, A. Guimond and R. Daigneault, Post unilateral lesion response biases modulate memory: crossed double dissociation of hemispheric specialisations, *Laterality* **14**(2) (2009), 122–164.

[6] P.R. Corlett, J.H. Krystal, J.R. Taylor and P.C. Fletcher, Why do delusions persist? *Front Hum Neurosci* **3** (2009), 12.

[7] D.C. Delis, E. Kaplan and J.H. Kramer, Delis-Kaplan Executive Function System (D–KEFS). Pearson Education, Inc. San Antonio, Tx (2001).

[8] O. Devinsky, Delusional misidentifications and duplications: right brain lesions, left brain delusions, *Neurology* **72**(1) (2009), 80–87.

[9] B. Dubois, A. Slachevsky, I. Litvan and B. Pillon, The FAB: a Frontal Assessment Battery at bedside, *Neurology* **55**(11) (2000), 1621–1626.

[10] S. Engelborghs, K. Maertens, G. Nagels, E. Vloeberghs, P. Marien, A. Symons, V. Ketels, S. Estercam, N. Somers and P.P. De Deyn, Neuropsychiatric symptoms of dementia: cross-sectional analysis from a prospective, longitudinal Belgian study, *Int J Geriatr Psychiatry* **20**(11) (2005), 1028–1037.

[11] A. Ferchiou, F. Schurhoff, E. Bulzacka, M. Mahbouli, M. Leboyer and A. Szoke, Mémoire de source – présentation générale et revue des études dans la schizophrénie, *Encephale* **36**(4) (2010), 326–333.

[12] A. Fotopoulou, False selves in neuropsychological rehabilitation: the challenge of confabulation, *Neuropsychol Rehabil* **18**(5–6) (2008), 541–565.

[13] A. Fotopoulou, M.A. Conway, S. Tyrer, D. Birchall, P. Griffiths and M. Solms, Is the content of confabulation positive? An experimental study, *Cortex* **44**(7) (2008), 764–772.

[14] R.J. Garoff-Eaton, E.A. Kensinger and D.L. Schacter, The neural correlates of conceptual and perceptual false recognition, *Learn Mem* **14**(10) (2007), 684–692.

[15] A. Gilboa, C. Alain, D.T. Stuss, B. Melo, S. Miller and M. Moscovitch, Mechanisms of spontaneous confabulations: a strategic retrieval account, *Brain* **129**(Pt 6) (2006), 1399–1414.

[16] A. Gilboa, C. Alain, Y. He, D.T. Stuss and M. Moscovitch, Ventromedial prefrontal cortex lesions produce early functional alterations during remote memory retrieval, *J Neurosci* **29**(15) (2009), 4871–4881.

[17] A. Gilboa and M. Verfaellie. Telling it like it isn't: the cognitive neuroscience of confabulation, *J Int Neuropsychol Soc* **16** (2010), 961–966.

[18] R. Glowinski, V. Payman and K. Frencham, Confabulation: a spontaneous and fantastic review, *Aust N Z J Psychiatry* **42**(11) (2008), 932–940.

[19] M. Harciarek and A. Kertesz, The prevalence of misidentification syndromes in neurodegenerative diseases, *Alzheimer Dis Assoc Disord* **22**(2) (2008), 163–169.

[20] W. Hirstein, Brain Fiction, Self-Deception and the Riddle of Confabulation, MIT Press, Cambridge MA, 2005.

[21] M.K. Johnson, S. Hashtroudi and D.S. Lindsay, Source monitoring, *Psychol Bull* **114**(1) (1993), 3–28.

[22] M. K. Johnson, M. O'Connor and J. Cantor, Confabulation, memory deficits, and frontal dysfunction, *Brain Cogn* **34**(2) (1997), 189–206.

[23] A. Kertesz, Frontotemporal dementia: a topical review, *Cogn Behav Neurol* **21**(3) (2008), 127–133.

[24] R.P. Kessels, H.E. Kortrijk, A.J. Wester and G.M. Nys, Confabulation behavior and false memories in Korsakoff's syndrome: role of source memory and executive functioning, *Psychiatry Clin Neurosci* **62**(2) (2008), 220–225.

[25] M.D. Kopelman, Varieties of confabulation and delusion, *Cogn Neuropsychiatry* **15**(1) (2010), 14–37.

[26] S.A. Kremen, O.E. Solis, J.S. Shapira, H.V. Vinters and M.F. Mendez, "Fantastic thinking" in pathologically proven Pick disease, *Cogn Behav Neurol* **23**(2) (2010), 130–134.

[27] H. Lamote, K.L. Tan and W.M. Verhoeven, Frontotemporale dementie bij een jonge vrouw met ogenschijnlijk schizofrenie, *Ned Tijdschr Geneeskd* **142**(35) (1998), 1962–1965.

[28] R. Langdon and M. Turner, Delusion and confabulation: overlapping or distinct distortions of reality? *Cogn Neuropsychiatry* **15**(1) (2010), 1–13.

[29] A.J. Larner, Delusion of pregnancy in frontotemporal lobar degeneration with motor neurone disease (FTLD/MND), *Behav Neurol* **19**(4) (2008), 199–200.

[30] F. Lebert, H. Lys, E. Haem and F. Pasquier, Syndrome démentiel dans les suites d'une bipolarité, *Encephale* **34**(6) (2008), 606–610.

[31] P. Lillo, B. Garcin, M. Hornberger, T.H. Bak and J.R. Hodges, Neurobehavioral features in frontotemporal dementia with amyotrophic lateral sclerosis, *Arch Neurol* **67**(7) (2010), 826–830.

[32] E. Lorente-Rovira, J. L. Santos-Gomez, M. Moro, J. M. Villagran and P. J. McKenna, Confabulation in schizophrenia: a neuropsychological study, *J Int Neuropsychol Soc* **16**(6), 1018–1026.

[33] C.T. Loy, J.J. Kril, J.N. Trollor, M.C. Kiernan, J.B. Kwok, S. Vucic, G.M. Halliday and J.R. Hodges, The case of a 48 year-old woman with bizarre and complex delusions, *Nat Rev Neurol* **6**(3) (2010), 175–179.

[34] A. Marchewka, A. Brechmann, A. Nowicka, K. Jednorog, H. Scheich and A. Grabowska, False recognition of emotional stimuli is lateralised in the brain: An fMRI study, *Neurobiol Learn Mem* **90**(1) (2008), 280–284.

[35] A. Marchewka, K. Jednorog, A. Nowicka, A. Brechmann and A. Grabowska, Grey-matter differences related to true and false recognition of emotionally charged stimuli – a voxel based morphometry study, *Neurobiol Learn Mem* **92**(1) (2009), 99–105.

[36] M.F. Mendez, J.S. Shapira, R.J. Woods, E.A. Licht and R.E. Saul, Psychotic symptoms in frontotemporal dementia: prevalence and review, *Dement Geriatr Cogn Disord* **25**(3) (2008), 206–211.

[37] M.F. Mendez and I.A. Fras, the false memory syndrome: Experimental studies and comparison to confabulations, *Med hypotheses* (2011) *in press*.

[38] B.L. Miller, L. Chang, I. Mena, K. Boone and I.M. Lesser, Progressive right frontotemporal degeneration: clinical, neuropsychological and SPECT characteristics, *Dementia* **4**(3–4) (1993), 204–213.

[39] S. Moritz, T.S. Woodward, J.C. Whitman and C. Cuttler, Confidence in errors as a possible basis for delusions in schizophrenia, *J Nerv Ment Dis* **193**(1) (2005), 9–16.

[40] M. Moscovitch and B. Melo, Strategic retrieval and the frontal lobes: evidence from confabulation and amnesia, *Neuropsychologia* **35**(7) (1997), 1017–1034.

[41] L. Nahum, R. Ptak, B. Leemann and A. Schnider, Disorientation, confabulation, and extinction capacity: clues on how the brain creates reality, *Biol Psychiatry* **65**(11) (2009), 966–972.

[42] D. Neary, J.S. Snowden, L. Gustafson, U. Passant, D. Stuss, S. Black, M. Freedman, A. Kertesz, P.H. Robert, M. Albert, K. Boone, B.L. Miller, J. Cummings and D.F. Benson, Frontotemporal lobar degeneration: a consensus on clinical diagnostic criteria, *Neurology* **51**(6) (1998), 1546–1554.

[43] Z. Nedjam, E. Devouche and G. Dalla Barba, Confabulation, but not executive dysfunction discriminate AD from frontotemporal dementia, *Eur J Neurol* **11**(11) (2004), 728–733.

[44] R. Omar, E.L. Sampson, C.T. Loy, C.J. Mummery, N.C. Fox, M.N. Rossor and J.D. Warren, Delusions in frontotemporal lobar degeneration, *J Neurol* **256**(4) (2009), 600–607.

[45] E. Reischle, K. Sturm, G. Schuierer and B. Ibach, Ein Fall von schizophrenieformer Störung bei frontotemporaler Demenz (FTD), *Psychiatr Prax* **30**(Suppl 2) (2003), 78–82.

[46] A. Schnider, C. von Daniken and K. Gutbrod, The mechanisms of spontaneous and provoked confabulations, *Brain* **119**(Pt 4) (1996), 1365–1375.

[47] H. Seelaar, K.Y. Klijnsma, I. de Koning, A. van der Lugt, W.Z. Chiu, A. Azmani, A.J. Rozemuller and J.C. van Swieten, Frequency of ubiquitin and FUS-positive, TDP-43-negative frontotemporal lobar degeneration, *J Neurol* **257**(5), 747–753.

[48] D. Tang-Wai, P. Lewis, B. Boeve, M. Hutton, T. Golde, M. Baker, J. Hardy, V. Michels, R. Ivnik, C. Jack and R. Petersen, Familial frontotemporal dementia associated with a novel presenilin-1 mutation, *Dement Geriatr Cogn Disord* **14**(1) (2002), (2010), 13–21.

[49] M.C. Tartaglia, A. Kertesz and L.C. Ang, Delusions and hallucinations in frontotemporal dementia: a clinicopathologic case report, *Cogn Behav Neurol* **21**(2) (2008), 107–110.

[50] J.C. Thompson, C.L. Stopford, J.S. Snowden and D. Neary, Qualitative neuropsychological performance characteristics in frontotemporal dementia and Alzheimer's disease, *J Neurol Neurosurg Psychiatry* **76**(7) (2005), 920–927.

[51] M. Turner and M. Coltheart, Confabulation and delusion: a common monitoring framework, *Cogn Neuropsychiatry* **15**(1) (2010), 346–376.

[52] M.S. Turner, L. Cipolotti and T. Shallice. Spontaneous confabulation, temporal context confusion and reality monitoring: a study of three patients with anterior communicating artery aneurysms, *J Int Neuropsychol Soc* **16** (2010), 984–994.

[53] M.S. Turner, L. Cipolotti, T.A. Yousry and T. Shallice, Confabulation: damage to a specific inferior medial prefrontal system, *Cortex* **44**(6) (2008), 637–648.
[54] M.S. Turner, J.S. Simons, S.J. Gilbert, C.D. Firth and P.W. Burgess, Distinct roles for lateral and medial rostral prefrontal cortex in source monitoring of perceived and imagined events, *Neuropsychologia* **46** (2008), 1442–1453.
[55] H. Urwin, K.A. Josephs, J.D. Rohrer et al., FUS pathology defines the majority of tau- and TDP-43-negative frontotemporal lobar degeneration, *Acta Neuropathol* **120**(1) (2010), 33–41.
[56] F. Vanderzeypen, J.C. Bier, C. Genevrois, J. Mendlewicz and F. Lotstra, Frontal dementia or dementia praecox? A case report of a psychotic disorder with a severe decline, *Encephale* **29**(2) (2003), 172–180.
[57] D. Velakoulis, M. Walterfang, R. Mocellin, C. Pantelis, B. Dean and C. McLean, Abnormal hippocampal distribution of TDP-43 in patients with-late onset psychosis, *Aust N Z J Psychiatry* **43**(8) (2009), 739–745.
[58] D. Velakoulis, M. Walterfang, R. Mocellin, C. Pantelis and C. McLean, Frontotemporal dementia presenting as schizophrenia-like psychosis in young people: clinicopathological series and review of cases, *Br J Psychiatry* **194**(4) (2009), 298–305.
[59] J.L. Waddington, H.A. Youssef, M.A. Farrell and J. Toland, Initial 'schizophrenia-like' psychosis in Pick's disease: case study with neuroimaging and neuropathology, and implications for frontotemporal dysfunction in schizophrenia, *Schizophr Res* **18**(1) (1995), 79–82.
